# Plant species richness and community assembly along gradients of elevation and soil nitrogen availability

**DOI:** 10.1093/aobpla/plaa014

**Published:** 2020-04-15

**Authors:** Taro Ohdo, Koichi Takahashi

**Affiliations:** 1 Graduate School of Science and Technology, Shinshu University, Asahi, Matsumoto, Japan; 2 Department of Biology, Faculty of Science, Shinshu University, Matsumoto, Japan; 3 Institute of Mountain Science, Shinshu University, Asahi, Matsumoto, Japan

**Keywords:** Community assembly, community structure, functional traits, leaf traits, niche differentiation, plant height, trait distribution

## Abstract

Environmental filters affect community assembly through the functional traits of species. However, the process of community assembly remains unclear because of the complex interactions among the many biotic and abiotic factors. This study aimed to examine the community assembly process of vascular plants along gradients of elevation (45‒2500 m a.s.l.) and soil nitrogen availability. This study examined the trait distribution patterns of four functional traits (plant height, leaf area, specific leaf area and leaf nitrogen concentration) of vascular plants (trees, herbs and ferns) in central Japan, using null model testing. The number of species decreased and increased at high elevations for tree species and herb and fern species, respectively. The numbers of both tree species and herb and fern species were positively correlated with soil nitrogen availability. Community-weighted means (CWMs) of the four traits decreased with elevation. CWMs and ranges of the three leaf traits were positively correlated with soil nitrogen availability. The community-weighted variance of plant height was higher at higher elevations, indicating that niche differentiation of vertical stratum in habitats with a low canopy was important for community assembly. This study suggests that severe climatic conditions reduce the number of tree species and the canopy height at high elevations, leading to increases in the number of herb and fern species due to increased light intensity at the forest floor. The elevational change of leaf traits also indicates the change of adaptive leaf traits. It further suggests that lower nitrogen availability decreases the number of tree, herb and fern species by excluding those species with leaf traits unsuited to lower nitrogen availability. Therefore, community structure is most likely regulated by both elevation and soil nitrogen availability.

## Teaser

This study examined the community assembly process of vascular plants along gradients of elevation (45‒2500 m) and soil nitrogen availability by using trait distribution patterns of four functional traits. This study suggests that severe climatic conditions reduce the number of tree species and the canopy height at high elevations, leading to increases in the number of herb and fern species due to increased light intensity at the forest floor. It further suggests that lower nitrogen availability decreases the number of tree, herb, and fern species by excluding those species with leaf traits unsuited to lower nitrogen availability.

## Introduction

Understanding community structures such as species richness and species composition is a key area of study in community ecology, and is important for predicting the effects of climate change on plant communities (Laughlin *et al.* 2011; Dubuis *et al.* 2013; Pescador *et al.* 2015). However, the process of community assembly remains unclear because of the complex interactions among many biotic and abiotic factors in addition to dispersion and stochastic processes (Bernard-Verdier *et al.* 2012; HilleRisLambers *et al.* 2012; Hulshof *et al.* 2013; Liancourt *et al.* 2017).

Recently, the importance of environmental filters and interspecific competition on plant community assembly has been examined by exploring functional traits (Cornwell and Ackerly 2009; Katabuchi *et al.* 2012; Dubuis *et al.* 2013; Hulshof *et al.* 2013; Long *et al.* 2015; Qi *et al.* 2015; Luo *et al.* 2016). The environmental filter concept suggests that species cannot tolerate in a given environment unless they have the traits necessary for tolerating that environment (Kraft *et al.* 2015b). Therefore, the trait range of a plant community is narrower in more severe environmental conditions such as high elevation, soil nutrient and water limitation (Diaz *et al*. 1998; Weiher *et al.* 1998; Kraft *et al.* 2008; Cornwell and Ackerly 2009; Katabuchi *et al.* 2012; Takahashi and Tanaka 2016).

The effect of interspecific competition on community assembly is not clear, as compared with the environmental filter, as above described. The limiting similarity hypothesis posits that species exclude ecologically similar species if there is severe interspecific competition. The trait frequency distribution of a particular trait in a plant community is more dispersed if niche differentiation is greater (Weiher *et al.* 1998; Kraft *et al.* 2008; Cornwell and Ackerly 2009; Katabuchi *et al.* 2012). Therefore, more intense interspecific competition diversifies the trait frequency distribution of a particular trait (i.e. giving a flatter distribution) more than random distribution by the exclusion of ecologically similar species (Bernard-Verdier *et al.* 2012; Chauvet *et al.* 2017). On the other hand, trait values of a particular trait may converge in conditions of intense interspecific competition (Grime 2006; Gerhold *et al.* 2013). For example, competition for light is one-sided or asymmetric competition (Weiner 1990; Takahashi 1996; Takahashi *et al.* 2003), i.e. larger plants acquire disproportionately greater light resources relative to their size. Therefore, superior (i.e. tall) species suppress inferior (i.e. short) species, especially in forest ecosystems with well-developed canopy structures. In fact, the biomass of understorey vegetation is negatively correlated with that of overstorey vegetation in forests (Alaback 1982). Therefore, the frequency distribution of trait values for a certain trait shows more convergence and a narrower trait range than would be the case with random distribution, because the plant community is assembled mainly by species with competitively superior traits, i.e. competition itself acts as a biotic filter (Grime 2006; Navas and Violle 2009; Dwyer *et al.* 2015). In this way, interspecific competition affects the frequency distribution of trait values differently (i.e. divergence or convergence), indicating that the role of interspecific competition on the community assembly process is unclear.

Generally, the number of plant species and canopy height decrease at higher elevations and less fertile sites (Tilman and Pacala 1993; Bhattarai and Vetaas 2003; Cornwell and Grubb 2003; Sánchez-González and López-Mata 2005; Miyajima and Takahashi 2007; Miyajima *et al.* 2007; Zhang *et al.* 2016), indicating changes in community assembly processes along environmental gradients. For example, nitrogen in particular is one of the most important soil nutrients which act as environmental filter. The effects of environmental filters on community assembly are greater in more stressful environmental conditions (Katabuchi *et al.* 2012; Spasojevic and Suding 2012). Therefore, it is expected that the effect of environmental filters on trait distributions will be greater at higher elevations and sites with less available nitrogen, and that they will restrict the number of species and the range of trait distributions. Conversely, interspecific competition is intense in established forests at low elevations and at sites where nitrogen is freely available and productivity is high, and this can also affect trait variances. Furthermore, the density of small plants such as tree saplings, herb and fern species increases in high elevations because of the undeveloped canopy structure with short stand height (Miyajima and Takahashi 2007). Many researchers investigated only trees larger than a certain size in forests to investigate community assembly processes from the viewpoint of functional traits. However, there are only a few studies that examined all vascular plant species such as tree, herb and fern species along gradients of elevation and soil nitrogen availability (Luo *et al*. 2019*a*, *b*). Investigation of all vascular plant species is indispensable for understanding changes of community assembly processes along environmental gradients.

The choice of functional traits is important for the effective analysis of plant community assembly processes. Westoby (1998) proposed a scheme of plant ecology strategies from viewpoints of leaf, plant height and seed mass. Measurements of plant height and leaf traits are easier than seed mass, and are appropriate for comparison among many plant species along environmental gradients. Therefore, this study focussed on plant height and three leaf traits, i.e. plant height, leaf area of individual leaves, specific leaf area (SLA) and leaf nitrogen concentration (N_mass_). First, plant height is related to the acquisition of light resources. Second, the leaf area of individual leaves relates to the acquisition of light energy and to water balance (Pérez-Harguindeguy *et al.* 2013). Finally, SLA and N_mass_ relate to leaf carbon economy and are also correlated with leaf longevity (Chabot and Hicks 1982; Koike 1988; Wright *et al.* 2004). Therefore, these four plant functional traits (plant height, leaf area, SLA and N_mass_) are all important traits that are closely related to community assembly processes.

This study analysed the effects of environmental filters and biotic interactions on the community assembly process along environmental gradients of elevation (45‒2500 m above sea level) and soil nitrogen availability, in central Japan. Vegetation largely changes along this elevational gradient from lowland hardwood forests to alpine dwarf pine scrubs. Specifically, we attempted to address the following four hypotheses:

(1) Plant height and the number of tree species decreases at higher elevations with more severe climatic stress, which increases the number of forest understorey plant species (herbs and ferns) by increasing light availability.
(2) The number of species decreases at sites where less nitrogen is available by exclusion of species with unsuitable leaf traits (large leaf area, high SLA and N_mass_).
(3) Community-weighted means of the three leaf traits (leaf area, SLA and N_mass_) change along the elevational gradient because of the change of climatic conditions.
(4) Strong interspecific competition increases the convergence of frequency distribution of plant height in low elevations with developed canopy structures by exclusion of small understorey plant species.

To test these hypotheses, we examined vegetation and trait distribution patterns of four plant functional traits (plant height, leaf area, SLA and N_mass_) in vascular plants (trees, herbs and ferns) along an elevational gradient using null model testing.

## Methods

### Study site

This study was conducted along an elevational gradient (45‒2500 m a.s.l.) on the Pacific side of central Japan. The part of the study site with the highest elevation (2500 m a.s.l.) was near the summit of Mount Norikura (3026 m a.s.l., N36°06′, E137°33′). Mean annual temperatures ranged from 16.0 °C (45 m a.s.l.) to ‒0.7 °C (2500 m a.s.l.) during 2017. Mean annual precipitation ranged from 1420 mm (45 m a.s.l.) to 2691 mm (2500 m a.s.l.) during 30 years, estimated by Land, Infrastructure, Transport and Tourism (http://nlftp.mlit.go.jp/ksj/gml/datalist/KsjTmplt-G02.html).

The vegetation in central Japan comprises evergreen and deciduous hardwood forests between 0 and 300 m a.s.l.; temperate conifer and hardwood mixed forests between 300 and 600 m a.s.l.; deciduous hardwood forests between 600 and 1600 m a.s.l.; subalpine coniferous forests between 1600 and 2500 m a.s.l.; and dwarf pine scrubs between 2500 and 3000 m a.s.l. (Ohsawa 1995; Miyajima *et al.* 2007). Some deciduous hardwood tree species are also found distributed as subordinate species between 1600 and 3000 m a.s.l. (Miyajima *et al.* 2007).

### Vegetation survey

Vegetation was investigated at 13 different elevation levels over three summers, between 2015 and 2017, to examine elevational changes in community structures. The elevations selected were 45, 340, 385, 800, 980, 1100, 1450, 1600, 1800, 2100, 2200, 2350 and 2500 m a.s.l. For the purposes of this study, two vegetation layers were defined: the overstorey layer (more than 1.3 m tall) and the understorey layer (less than 1.3 m tall). To investigate the abundance of tree species more than 1.3 m tall, two plots (5 × 50 m) were established at most elevations (the exceptions were the 340 m and 2500 m a.s.l. elevation levels, which each had just one plot). The total number of plots was 24. Each plot was divided into 10 quadrats of 5 × 5 m. To investigate the abundance of all vascular plant species (herbs, ferns and trees) less than 1.3 m tall, a 1 × 1 m quadrat was set at the upper right corner of each 5 × 5 m quadrat in each 5 × 50 m plot. Species were identified and the percentage cover was determined for each tree species more than 1.3 m tall in each 5 × 5 m quadrat and for each vascular plant species less than 1.3 m tall at each 1 × 1 m quadrat. For plant nomenclature we followed Ohashi *et al.* (2015) for tree and herb species and the Editorial Board of Flora of Nagano Prefecture (1997) for fern species.

The study site comprised secondary forest at 45, 340 and between 800 and 1400 m a.s.l., and natural forest at 385, and between 1600 and 2500 m a.s.l. (Biodiversity Centre of Japan, http://gis.biodic.go.jp/webgis/index.html). Ideally, we would have included an investigation of vegetation in only old-growth natural forest along the elevational gradient for the analysis. However, there are virtually no old-growth natural forests at low elevations in Japan. Although the vegetation at 385 m a.s.l., part of the study site at a low elevation, consisted of natural forest, this site was an exception because it included a reserved forest (Takahashi *et al.* 2018). However, the vegetation in the secondary forest at the other low elevation sites had already considerably developed because the natural vegetation here had remained unchanged for at least the last 50 years (Biodiversity Centre of Japan, http://gis.biodic.go.jp/webgis/index.html). Therefore, we used vegetation data from these secondary forest areas for the analysis in this study.

### Environmental data

We obtained estimated climate data parameters for the 13 elevation levels–mean annual temperature (°C), total annual rainfall (mm), and mean annual wind velocity (m/s)–from databases held by the Japanese Ministries of the Environment (http://www.env.go.jp/earth/ondanka/windmap/map.html) and Land, Infrastructure, Transport and Tourism (http://nlftp.mlit.go.jp/ksj/gml/datalist/KsjTmplt-G02.html).

Soil samples were collected from the 13 elevation levels over two days (3 and 7 August 2017). Approximately 300 cm^3^ of soil was collected from the top 5 cm of the B horizon at each plot using a shovel. The soil samples were immediately placed into plastic bags to prevent them from drying out and were taken back to the laboratory. The soil samples were crushed and sieved using a 2 mm sieve. Inorganic nitrogen was extracted from the soil samples by distilled water after air-drying the soil. The concentration of nitrate (NO_3_^-^), ammonium (NH_4_^+^) and potassium (K) were determined using a reflectometer (RQflex plus 10, Merck Ltd, Germany). The concentrations of NO_3_^-^ and NH_4_^+^ were converted to concentrations of NO_3_-N and NH_4_-N, respectively. Total carbon (C) and nitrogen (N) were measured using an elemental analyser (Thermo Finnigan Flash EA 1112, Thermo Fisher Scientific Inc., MA), and the C/N ratio was determined. The C/N ratio is an indicator of nitrification rate (i.e. the amount of nitrogen in the form of inorganic nitrate available for plants) because of the negative correlation between the C/N ratio and the nitrification rate (Luizao *et al.* 2004; Soethe *et al.* 2008).

### Plant functional traits

For this study, we selected four functional traits, namely plant height, leaf area of individual leaves, SLA and N_mass_. We measured the height of three to five individual plants of average height for each tree species that formed part of the overstorey layer in each plot (5 × 50 m) by eye because the top of the canopy was almost 20 m tall. We also measured the approximate height of three to five individual plants for each vascular plant species that formed part of the understorey layer in each plot, using a measuring pole. We sampled three to five mature current-year leaves from at least three individuals at each elevation level. Pérez-Harguindeguy *et al.* (2013) recommended at least ten and five individuals for measurements of plant height and leaf traits, respectively. Thus, the number of individuals sampled in this study was somewhat less than the recommended minimum number, especially for plant height. However, as practical problem, it was difficult to find 10 individuals within a plot for each species, especially for minor species.

The sampled leaves were taken back to the laboratory and scanned using a flatbed scanner. The leaf area of individual leaves was measured using the free software ImageJ (ver. 1.46) (http://rsbweb.nih.gov/ij/download.html), then the dry mass of leaves was weighed after oven-drying at 80 ℃ for 48 h. Leaf petiole was not included for the measurement of leaf area and dry mass. The SLA was calculated by dividing leaf area by dry mass. The leaf samples were ground into a powder and N_mass_ was measured using an elemental analyser (Thermo Finnigan Flash EA 1112, Thermo Fisher Scientific Inc., MA). The average of three to five trait values for each trait was calculated for each species at each elevation level.

### Null model analysis

We created null models to examine the filtering of trait values and the shaping of trait frequency distributions for the four functional traits at each plot, according to the null model analysis described by Bernard-Verdier *et al.* (2012). A null model 1 (NM1), incorporating an interspecific variation of each trait, was created to analyse the environmental filtering of trait values. The NM1 analysis was done to test whether species occurrences are distributed among plots randomly in terms of trait values. We created 999 random communities for the NM1 analysis by repeating the following three steps. (i) A mean trait value from a particular elevation was randomly selected from the multiple elevation levels for each functional trait of each species because many species were observed at multiple elevations, and that selected trait value was assigned to the species. Then, we created a list of species abundance at the 13 elevation levels (i.e. the regional species pool) and a list of the four functional trait values for each of the observed species at the 13 elevation levels. (ii) For each plot, we made a random community by choosing the same number of species observed at the plot from the list of the regional species pool, with the probability of a species being chosen proportional to its regional abundance (Bernard-Verdier *et al.* 2012). (iii) A range (maximum value–minimum value) was calculated for each functional trait at the random community. After creating 999 random communities by repeating the step 1 to 3, a mean value and the standard deviation of the range were calculated for each functional trait at each plot. If the observed range is smaller than expected by the NM1, it indicates the trait filtering. In short, the NM1 makes a random community by shuffling trait values of species in a target plot with those in the regional species pool, and calculates abundance-weighted trait value from 999 random communities.

A null model 2 (NM2) was created to examine if trait frequency distribution of each trait was convergence or divergence at each plot (i.e. effects of interspecific competition). We created 999 random communities for the NM2 analysis. Each random community was made as follows. A list of species abundance and the four functional trait values was made for each plot. The species abundance was shuffled, then the community-weighted mean (CWM) and the community-weighted variance (CWV) was calculated for each trait as follows:

$$\begin{array}{l} CWM=\sum (a_{i}\times t_{i}) \end{array}$$

$$\begin{array}{l} CWV=\sum \left(a_{i}\times \;{\left(t_{i}-CWM\right)}^{2}\right) \end{array}$$

where *a*_*i*_ is the relative coverage of a species *i*, and *t*_*i*_ is the trait value of a species *i*. After creating 999 random communities, the mean value and standard deviation of CWV was calculated for each plot. If the observed CWV is lower than expected by the NM2, it indicates the convergence towards a common trait value (Bernard-Verdier *et al.* 2012).

The standardised effect size (SES) can be used to express the direction and size of each metric (range and CWV), and was calculated as follows:

$$\begin{array}{l} SES=\frac{I_{obs}-I_{null}}{\sigma_{null}}\; \end{array}$$

where *I*_obs_ is the observed value, and *I*_null_ and σ _null_ are the mean and the standard deviation of the 999 random communities, respectively. The range was used as an index of environmental filters. An environmental filter is expected to reduce the range of observed trait values below the null expectation. Therefore, we examined whether the SES of a range was significantly lower than the null expectation using a one-tailed test. CWV was used as an index for the shaping of trait frequency distributions. It is thought that a trait frequency distribution undergoes convergence or divergence if many species have similar trait values for a certain functional trait, or many species have different trait values for a certain functional trait, respectively. Therefore, we examined whether the SES of the CWV was significantly lower (convergence) or higher (divergence) than the null expectation using a two-tailed test. The distribution pattern of SES values for 999 random communities showed a normal distribution (mean = 0, standard deviation = 1). The 95 % confidence interval is expected to be more negative than ‒1.65 and within ±1.96 for the one-tailed and two-tailed tests, respectively. Observed SES values outside the 95 % confidence intervals are considered to be significantly different from the null expectation. We performed the null model testing using a program coded in C++.

### Relationships between plant communities and environmental factors

It is possible that climatic factors (wind velocity, rainfall and temperature) and soil factors (NO_3_-N, NH_4_-N, K, total N, total C, and C/N ratio) correlate with each other to some extent. Therefore, we used principle component analysis to reduce the number of variables. Spearman’s rank correlation test was performed whether principal component scores correlated with climatic and soil factors. The simultaneous autoregressive (SAR) model was also conducted to analyse whether principal component scores correlated with the number of species and three trait metrics, CWM, range (SES) and CWV (SES) of each functional trait for avoidance of spatial autocorrelations. We performed the statistical analysis using the free software package R (R Core Team 2020). We used the *spdep* package (Bivand 2019) to conduct the SAR model.

## Results

### Environmental conditions and number of species

Climate and soil factors could be summarized into two principle components (PC) **[see****Supporting Information—Fig. S1****]**. PC1 and PC2 could explain 64.8 and 18.2 % of the total variance, respectively. PC1 was negatively correlated with mean annual temperature (*R* = −0.872, *P* < 0.001, Table 1) and positively correlated with mean annual wind velocity (*R* = 0.790, *P* < 0.001), total annual rainfall (*R* = 0.905, *P* < 0.001), concentration of NH_4_-N (*R* = 0.852, *P* < 0.001), potassium (*R* = 0.929, *P* < 0.001), total nitrogen (*R* = 0.901, *P* < 0.001) and total carbon (*R* = 0.923, *P* < 0.001). Although ‘elevation’ was not included as a variable in the principal component analysis, PC1 was positively correlated with elevation (*R* = 0.863, *P* < 0.001). Therefore, PC1 expressed elevational changes in climatic conditions and some soil factors. PC2 was negatively correlated with the concentration of NO_3_-N (*R* = ‒0.886, *P* < 0.001, Table 1) and positively correlated with the C/N ratio (*R* = 0.720, *P* < 0.01). Therefore, PC2 indicated low nitrogen availability for plants due to decreased NO_3_-N.

**Table 1.** Spearman’s rank correlation coefficients between the two principal components (PC1 and PC2) and environmental variables.

Variable	PC1	PC2
Climate factors		
Mean annual wind velocity	0.790***	0.083
Total annual rainfall	0.905***	‒0.171
Mean annual temperature	‒0.872***	0.231
Soil factors		
NO3-N concentration	0.256	‒0.886***
NH_4_-N concentration	0.852***	0.385
K concentration	0.929***	‒0.126
Total nitrogen concentration	0.901***	‒0.170
Total carbon concentration	0.923***	0.121
C/N ratio	0.445	0.720**

*P < 0.05, **P < 0.01, ***P < 0.001.

**Figure 1.**
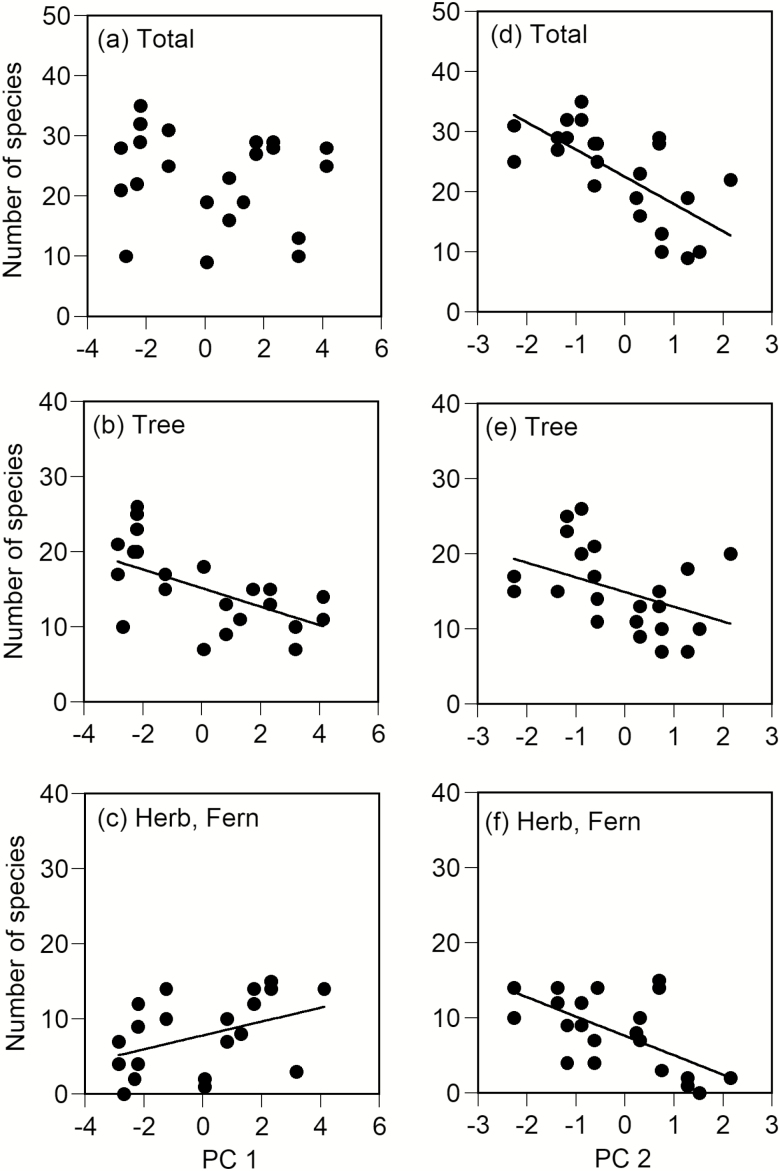
Relationships among the first and second principal component scores (PC1 and PC2) and the number of species for all vascular plant species (a, d), tree species (b, e) and non-tree species (c, f). Regression lines were drawn for significant correlations only (*P <* 0.05 by the SAR model).

A total of 234 species were found along the elevational gradient **[seeSupporting Information—Table S1]**. Of these 234 species, the number of tree, herb and fern species was 164, 58 and 12 species, respectively. Fern and herb species were grouped together because there were only 12 species of ferns. The total number of species was not correlated with PC1 (*P* = 0.403, Fig. 1a). Although the number of tree species significantly decreased with PC1 (*P* < 0.001, Fig. 1b), the number of herb and fern species significantly increased (*P* < 0.05, Fig. 1c), indicating different responses to elevation among tree species and herb and fern species. Conversely, PC2 was negatively correlated with the total number of species (*P* < 0.001, Fig. 1d), the number of tree species (*P* < 0.05, Fig. 1e), and the number of herb and fern species (*P* < 0.001, Fig. 1f). This indicated that there were fewer species at sites where less nitrogen was available, irrespective of taxonomic group.

### Functional traits

**Figure 2.**
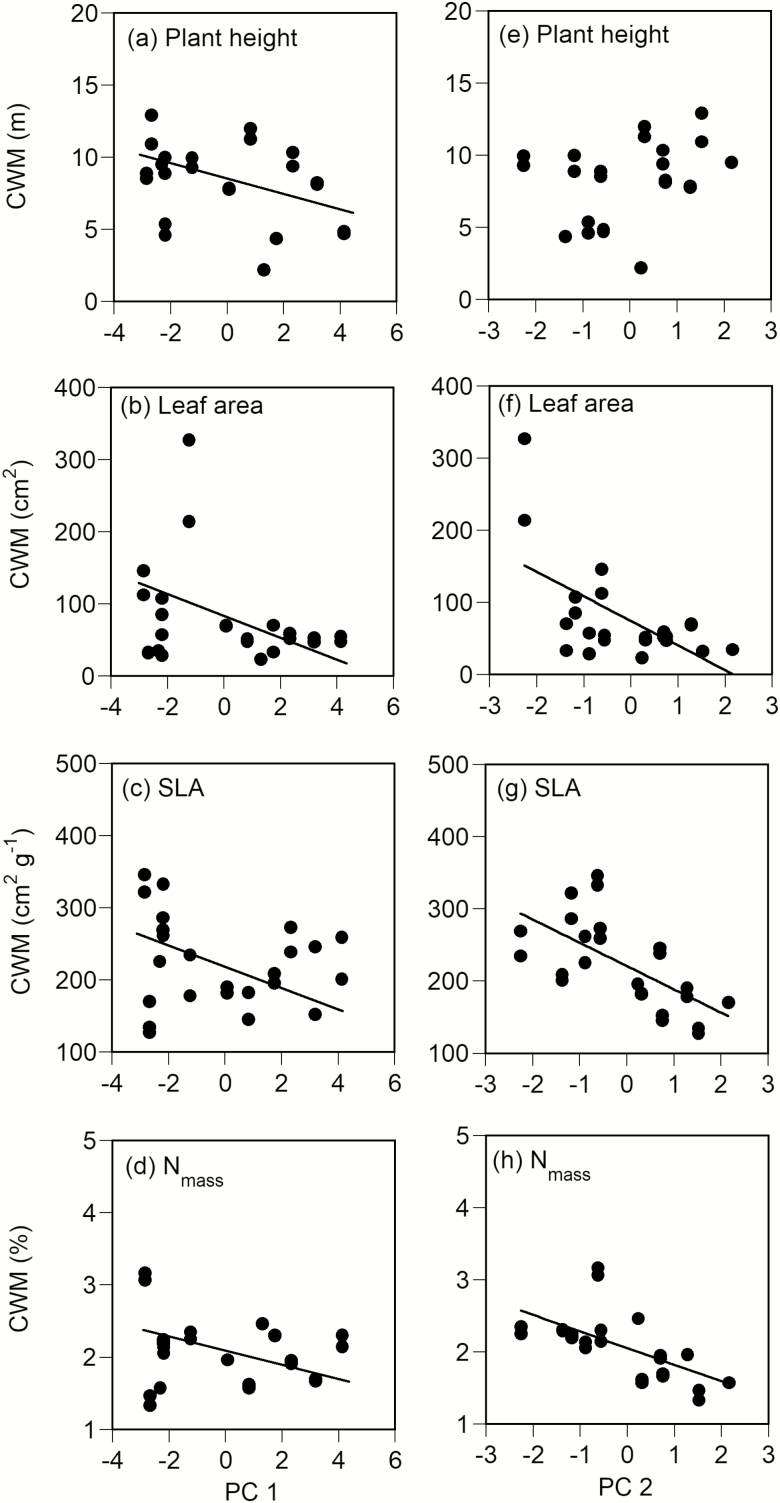
Relationships among the first and second principal component scores (PC1 and PC2) and the community-weighted means (CWM) for plant height (a, e), leaf area of individual leaves (b, f), specific leaf area (SLA) (c, g) and leaf nitrogen concentration (N_mass_) (d, h). Regression lines were drawn for significant correlations only (*P <* 0.05 by the SAR model).

The four functional traits (plant height, leaf area, SLA and N_mass_) of the 234 species are listed in **Supporting Information— Tables S2‒S5**, respectively. The CWMs of plant height (*P* < 0.05) and three leaf traits (leaf area, SLA and N_mass_, *P* < 0.01 for each) significantly decreased with PC1 (Fig. 2a‒d). Although the CWM of plant height showed no significant correlation with PC2 (Fig. 2e), the three leaf traits (leaf area, SLA and N_mass_) significantly decreased negative correlations with PC2 (*P* < 0.001 for each, Fig. 2f‒h). Therefore, the plant height decreased in high elevations and the three leaf traits were less assimilative characteristics at high elevations and sites where less nitrogen was available.

**Figure 3.**
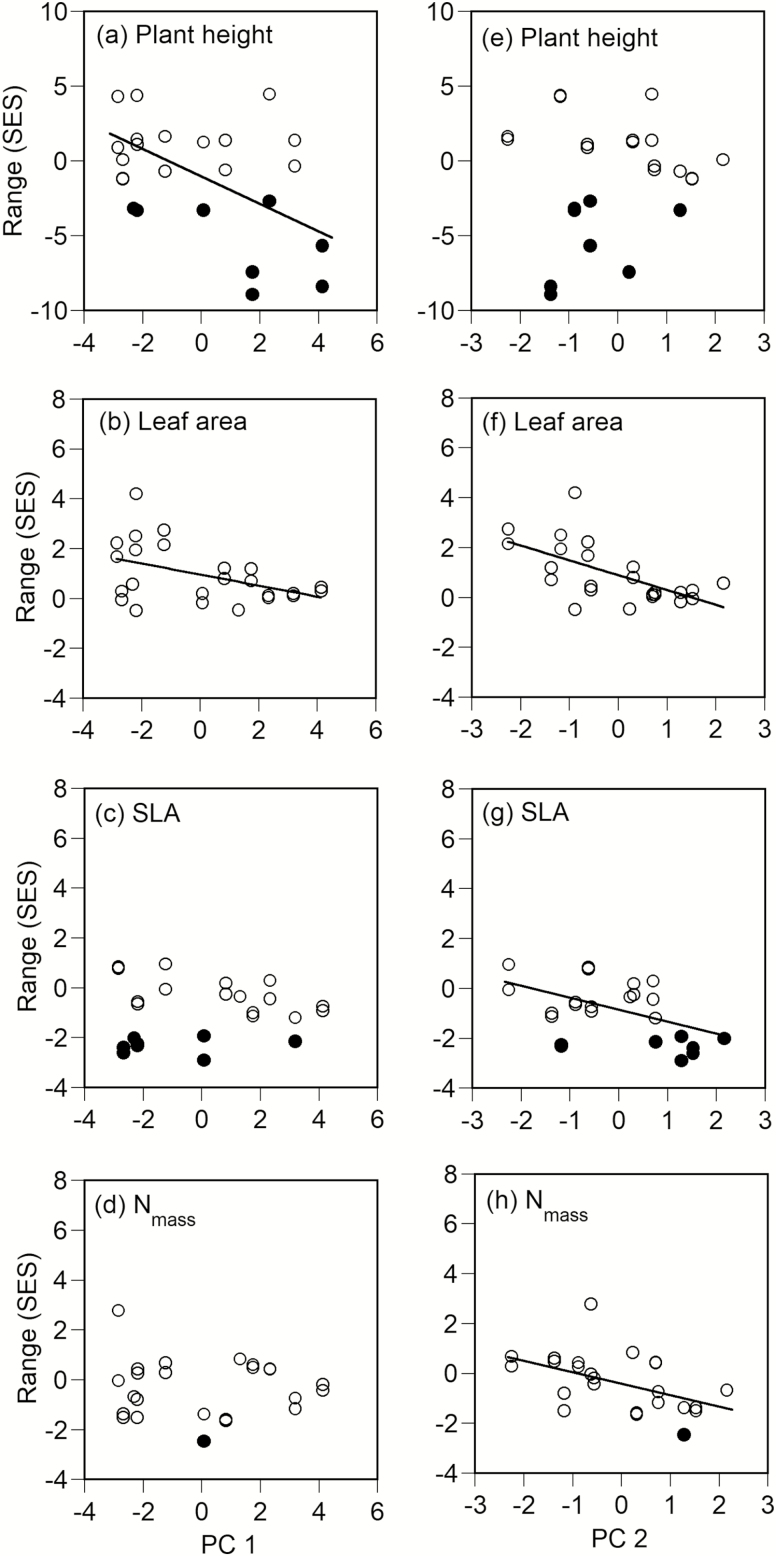
Relationships among the first and second principal component scores (PC1 and PC2) and ranges (SES) for plant height (a, e), leaf area of individual leaves (b, f), specific leaf area (SLA) (c, g) and leaf nitrogen concentration (N_mass_) (d, h). Solid circles represent significantly lower values than the 95 % confidence intervals by the one-tailed test. Regression lines were drawn for significant correlations only (*P <* 0.05 by the SAR model).

The ranges of plant height and leaf area significantly decreased with PC1 (*P* < 0.05 for each, Fig. 3a, b), while those of SLA and N_mass_ were not correlated with PC1 (Fig. 3c, d). Although the range was significantly smaller than the null expectation at one plot for N_mass_ and some plots for plant height and SLA, these plots varied from low to high PC1 values (Fig. 3a, c, d). The range of plant height was also not correlated with PC2 (Fig. 3e). However, the ranges of the three leaf traits (leaf area, SLA and N_mass_) significantly decreased with PC2 (at least *P* < 0.01, Fig. 3f‒h). Therefore, the ranges of trait values for plant height and leaf area and those for the three leaf traits were narrower at high elevations and sites where less nitrogen was available, respectively.

**Figure 4.**
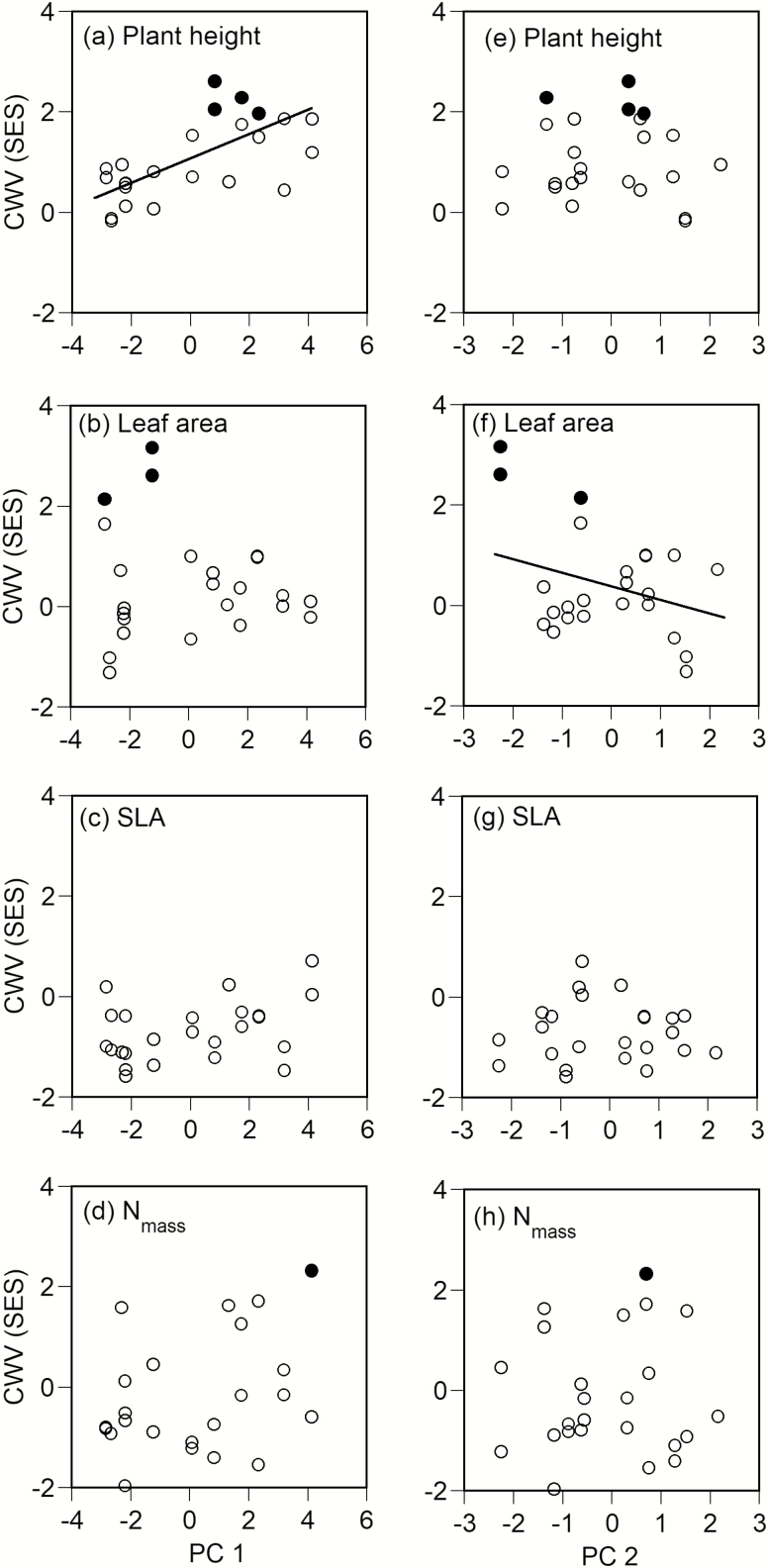
Relationships among the first and second principal component scores (PC1 and PC2) and CWV (SES) for plant height (a, e), leaf area of individual leaves (b, f), specific leaf area (SLA) (c, g) and leaf nitrogen concentration (N_mass_) (d, h). Solid circles represent significantly lower or higher than the 95 % confidence intervals by the two-tailed test. Regression lines were drawn for significant correlations only (*P* < 0.05 by the SAR model).

The CWV of plant height significantly increased with PC1 (*P* < 0.001, Fig. 4a), i.e. the trait distribution was flatter at higher elevations. However, the CWVs of the three leaf traits (leaf area, SLA and N_mass_) were not correlated with PC1 (Fig. 4b‒d). The CWVs of three traits (plant height, SLA, and N_mass_) also showed no significant correlations with PC2 (Fig. 4e, g, h). However, the CWV of leaf area significantly decreased with PC2 (*P* < 0.05, Fig. 4f). Therefore, the leaf area of individual leaves was more convergent at sites where less nitrogen was available.

## Discussion

### Effects of elevation on community assembly

The number of tree species and the number of herb and fern species were negatively and positively correlated, respectively, with the PC1 that expressed elevation. Therefore, the total number of species was not found to decrease at high elevations. The number of tree species generally decreases or shows a hump-shaped pattern along elevational gradients (e.g. Yamada 1977; Ohsawa 1995; Bhattarai and Vetaas 2003; Sánchez-González and López-Mata 2005; Miyajima *et al.* 2007). The reduction in the number of tree species with increasing elevation, as well as latitude, at the global scale is closely related to reductions in primary productivity that are regulated by thermal conditions (Currie and Paquin 1987; Adams and Woodward 1989). Therefore, there is no doubt that thermal conditions greatly affected the number of tree species found along the elevational gradient examined in this study. However, disturbances may also affect elevational changes in the number of tree species. Although the canopy height is almost constant between 800 m and 2000 m a.s.l. on Mount Norikura, it largely decreases from 2200 m a.s.l. to the timberline (2500 m a.s.l.), as a result of disturbances due to strong winds and heavy snow (Miyajima and Takahashi 2007; Takahashi *et al.* 2012). The density of understorey individuals largely increases from 2200 m a.s.l. to the timberline, accompanying the reduction in canopy height (Miyajima and Takahashi 2007). The decrease in canopy height, or the lack of development of the canopy layer, increases the density of understorey individuals by increasing the light intensity at the forest floor (Alaback 1982). There have been reports that the number of understorey plant species is positively correlated with the light intensity at the forest floor in boreal forests in North America (Hart and Chen 2008; Reich *et al.* 2012). Therefore, it is thought that the increased light intensity at the forest floor due to the lack of canopy layer development led to the increased number of herb and fern species seen at high elevations in the present study. Furthermore, it is suggested that different factors affect the number of tree species compared with those that affect herb and fern species, for example large-scale climatic factors (e.g. temperature) tend to affect the number of tree species while small-scale factors (e.g. canopy structure) tend to affect the number of herb and fern species.

The canopy height decreases abruptly from 2200 m a.s.l. to the timberline (2500 m a.s.l.) on Mount Norikura, as described above. The CWM of plant height also significantly decreased with PC1 in this study. Plants of various heights were distributed below the low canopy at high elevations (i.e. the CWV was greater at higher elevations), probably because of the undevelopment of the canopy layer. Conversely, understorey vegetation was suppressed at low elevations by the well-developed canopy layer. Although interspecific competition tends to diversify a trait distribution by excluding ecologically similar species, according to the limiting similarity hypothesis (Kraft *et al.* 2008; Cornwell and Ackerly 2009; Katabuchi *et al.* 2012; Takahashi and Tanaka 2016), extreme one-sided competition for light may increase the convergence of plant height distribution at low elevations by excluding suppressed understorey species (Takahashi 2010). Therefore, the divergent distribution of plant height observed at high elevations results from less interspecific competition due to the undeveloped canopy layer rather than from extreme interspecific competition.

The CWMs of the three leaf traits (leaf area, SLA and N_mass_) decreased with PC1. These changes of the three leaf traits were probably caused by the dominance of evergreen conifers at high elevations because trait values of the three leaf traits are smaller in evergreen conifers than in hardwood species. In the elevational gradient (45‒2500 m a.s.l.) examined in this study, vegetation changed with increasing elevation from evergreen and deciduous hardwood forests (~300 m a.s.l.), to temperate conifer-hardwood mixed forests (~600 m a.s.l.), to deciduous hardwood forests (~1600 m a.s.l.), to subalpine evergreen coniferous forests (~2500 m a.s.l.) and to evergreen dwarf pine scrubs (~3000 m a.s.l.). In general, leaf longevity is negatively correlated with the maximum photosynthetic rate and positively correlated with the leaf construction cost (expediently, this is the inverse of SLA) (Kikuzawa 1991). The maximum photosynthetic rate is positively correlated with N_mass_ (Reich *et al.* 1992). The SLA and N_mass_ of deciduous trees with shorter leaf longevity is generally greater than that of evergreen trees (Takahashi and Miyajima 2008; Bai *et al.* 2015). The growth rate of plant species that have a greater SLA and N_mass_ tends to be higher and their leaf longevity is shorter (Reich *et al*. 1991, 1992). Conversely, slow-growing species have a competitive advantage compared with fast-growing species in severe environmental conditions, such as infertile soil conditions, drought and low temperatures (Chapin 1980; Wright *et al.* 2004). In fact, a positive correlation was found between the CWM of SLA and that of N_mass_ (*R* = 0.823, *P* < 0.001). Therefore, the elevational changes of SLA and N_mass_ reflect the trade-off between the assimilative capacity and leaf longevity.

Considering the two negative correlations of PC1 with the CWM and range of leaf area, it is likely that species with large leaf area decrease in high elevations. Coniferous species with small leaf area distribute in high elevations, but hardwood tree species with extreme large compound leaves (e.g. *Aesculus turbinate*) do not distribute in high elevations. A large leaf area of individual leaves can be advantageous in light acquisition. However, a large leaf area is disadvantageous through leaf susceptibility to strong winds in high elevations (Niklas 1996). Therefore, the decrease of CWMs of the three leaf traits (leaf area, SLA, N_mass_) and the range of leaf area in high elevations reflect the elevational changes of adaptive leaf traits.

### Effects of soil nitrogen availability on community assembly

Although PC2 was not correlated with total carbon and total nitrogen levels in this study, PC2 was positively correlated with the C/N ratio and negatively correlated with the concentration of nitrate nitrogen. The rate of nitrification decreases with increasing soil C/N ratio, even if total carbon and total nitrogen are high, because of the immobilisation of nitrogen (Tate 1995; Whitmore and Handayanto 1997; Bengtsson *et al.* 2003). Therefore, it is assumed that the availability of soil nitrogen for plants decreased as PC2 increased. Some studies have shown that the number of species is low in areas of forests where conditions are less fertile (Loucks 1962; Peet and Chistensen 1988; Cornwell and Grubb 2003). In the present study, low soil nitrogen availability is also suggested to be a limiting factor for the number of plant species, irrespective of the range of taxonomic groups.

Species which have a small leaf area, low SLA and low N_mass_ were dominant at sites where less nitrogen was available because the CWMs and ranges of these three leaf traits (leaf area, SLA and N_mass_) decreased with increasing PC2 (linked with the decrease in nitrogen availability). In particular, species with a small leaf area were dominant at sites where less nitrogen was available because the CWV of leaf area was negatively correlated with PC2. The maximum photosynthetic rate of leaves with low SLA and low N_mass_ is low at less fertile sites (Chapin 1980), because photosynthetic rate is positively correlated with SLA and N_mass_ (Reich *et al.* 1992). However, leaf longevity tends to be longer for leaves with small leaf area, low SLA and low N_mass_ (Reich *et al.* 1992; Ackerly and Reich 1999; Villar and Merino 2001). The longer retention of nutrients in plants due to increased leaf longevity is an adaptive trait in less fertile soil conditions, and decreases a plant’s nutrient demand (Chapin 1980). Therefore, we considered that the negative correlation among the CWMs and the ranges of these three leaf traits with PC2 was related to the exclusion of species that had leaf traits unsuited to lower nitrogen availability. In other words, the amount of soil nitrogen available for plants acted as an environmental filter for the community assembly.

## Conclusion

This study examined the community assembly process along gradients of elevation and soil nitrogen availability and suggested three important findings: (i) High climatic stress at high elevations reduces the number of tree species and the canopy height, which in turn increases the light intensity reaching the forest floor, resulting in an increase in the number of herb and fern species; (ii) The total number of plant species decreases at sites with lower nitrogen availability because the distribution of species with leaf traits unsuited to lower nitrogen availability is limited (i.e. nitrogen availability acts as an environmental filter); (iii) Community-weighted means of the three leaf traits (leaf area, SLA, N_mass_) decreased with elevations, indicating elevational changes of adaptive leaf traits; (iv) Strong interspecific competition decreased the variance of the frequency distribution of plant height in low elevations with developed canopy structure. In previous studies on community assembly, it was assumed that the shape of the trait frequency distribution of a given local community was determined by interspecific competition, after the trait distribution range had been determined from the regional species pool by environmental filters (Cornwell and Ackerly 2009; Bernard-Verdier *et al.* 2012; Chauvet *et al.* 2017). Based on this assumption, it is considered that environmental filters work more effectively on the regional species pool than interspecific competition (Kraft and Ackerly 2010). Furthermore, most previous studies that reported a reduction in the number of species through interspecific competition were conducted with narrow ranges of climate and vegetation (Gerhold *et al.* 2013; Dwyer *et al.* 2015). Under such conditions, the difference in the number of species among plant communities is considered to be small because of small differences in environmental conditions, so it may be the case that the greater the interspecific competition, the lower the number of species. Therefore, it is suggested that the number of species is regulated more by environmental filters than by interspecific competition in the present study, where community structures along a large gradient of elevation and soil nitrogen availability were examined. Our investigation of all vascular plant species (tree, herb and fern species) enabled to clarify the change of community assembly process along the environmental gradients. Climate change and atmospheric nitrogen deposition caused by human activities will affect elevational vegetation patterns by changing thermal and nutrient conditions (Fenn *et al.* 2003; Stevens *et al.* 2004; Lenoir *et al.* 2008; Takahashi 2014). The results of the study reported here will be useful for forecasting the impact of environmental changes on plant community structures.

This study analysed effects of environmental filtering and interspecific competition on the community assembly process along gradients of elevation and soil nitrogen availability by using the trait-based approach. However, recent works argued the following two points. First, it is impossible to distinguish whether environmental filtering or interspecific competition or a combination of both are determining the community assembly because both mechanisms can produce similar dispersion patterns of trait frequency distributions (e.g. convergence and divergence) (Kraft *et al.* 2015b; Cadotte and Tucker 2017). Second, multi-dimensional nature of plant functional traits is important on the community assembly (Laughlin and Wilson 2014; Kraft *et al.* 2015a). Although this study used the single-trait analysis under the assumption that environmental filtering and interspecific competition can be detectable by the metrics of frequency distribution patterns of each functional trait, further studies are necessary to reveal how the multi-dimensional nature of functional traits affects the community assembly along gradients of elevation and soil nitrogen availability.

## Supporting Information

The following additional information is available in the online version of this article—

**Table S1**. List of plant cover for each species at each elevation.

**Table S2**. List of plant height for each species at each elevation.

**Table S3**. List of leaf area for each species at each elevation.

**Table S4**. List of specific leaf area for each species at each elevation.

**Table S5**. List of nitrogen concentration for each species at each elevation.

**Figure S1.** Principal component (PC) analysis of 13 sites (circles) by climate and soil factors (arrows). Temperature, mean annual temperature; wind, mean annual wind velocity; rain, total annual rainfall; K, potassium concentration; C, total carbon concentration; N, total nitrogen concentration; C/N ratio, carbon to nitrogen ratio; NO_3_-N, nitrate nitrogen concentration; NH_4_-N, ammonium nitrogen concentration.

plaa014_suppl_supplementary_Table_S1

plaa014_suppl_supplementary_Table_S2

plaa014_suppl_supplementary_Table_S3

plaa014_suppl_supplementary_Table_S4

plaa014_suppl_supplementary_Table_S5

plaa014_suppl_supplementary_Fig_S1
