# Supplementary material for: Plant species richness and community assembly along gradients of elevation and soil nitrogen availability
Source: AoB Plants. 2020 Apr 15;12(3):plaa014. doi: 10.1093/aobpla/plaa014 (PMC7243275; doi:10.1093/aobpla/plaa014)
Supplement: plaa014_suppl_supplementary_Fig_S1 [file plaa014_suppl_supplementary_fig_s1.docx]

**Figure S1.** Principal component (PC) analysis of 13 sites (circles) by climate and soil factors (arrows). Temperature, mean annual temperature; wind, mean annual wind velocity; rain, total annual rainfall; K, potassium concentration; C, total carbon concentration; N, total nitrogen concentration; C/N ratio, carbon to nitrogen ratio; NO_3_-N, nitrate nitrogen concentration; NH_4_-N, ammonium nitrogen concentration.
